# Angiotensin II receptor blockers and risk of acute pancreatitis - a population based case–control study in Sweden

**DOI:** 10.1186/s12876-017-0595-8

**Published:** 2017-03-07

**Authors:** Tomas S. Bexelius, Rickard Ljung, Fredrik Mattsson, Yunxia Lu, Mats Lindblad

**Affiliations:** 1 Upper Gastrointestinal Research, Department of Molecular Medicine and Surgery, Karolinska Institutet, Stockholm, Sweden; 2 Department of Medical Epidemiology and Biostatistics, Karolinska Institutet, Box 281, S-171 77 Stockholm, Sweden; 3 Institute of Environmental Medicine, Karolinska Institutet, Stockholm, Sweden; 4 Program in Public Health, University of California, Irvine, CA USA; 5 Department of CLINTEC, Karolinska Institutet, Stockholm, Sweden

**Keywords:** Pancreas, Inflammation, Pharmacoepidemiology, Metabolic syndrome, Risk factors, Drugs, Hypertension, Prevention

## Abstract

**Background:**

Acute pancreatitis is a potentially lethal disease, with a rising incidence in the Western world. Yet, no pharmacological prevention or specific treatment for acute pancreatitis exists. Also, the connection with severity of acute pancreatitis is unknown. Experimental and epidemiological research suggests a protective effect of angiotensin II receptor blockers.

**Methods:**

During 2006 to 2008, we performed a nationwide case–control study on Swedish residents aged 40–84 years. First-time cases with acute pancreatitis were identified in the National Patient Register and data on dispensed prescriptions was retrieved from the Prescribed Drug Register. Controls were randomly selected from the general population in Sweden frequency-matched on sex, age, and calendar year. To estimate relative risk of acute pancreatitis, by degree of severity, among users of angiotensin II receptor blockers, as compared to non-users, we used multivariable logistic regression analysis to calculate odds ratios (OR) with 95% confidence interval (CI).

**Results:**

Among 6,161 cases of acute pancreatitis and 61,637 controls, current use of angiotensin II receptor blockers was followed by a decreased risk of acute pancreatitis, compared to non-users, adjusted OR 0 · 77 (95% CI 0 · 69–0 · 86). No protective association, but an increased risk was found for users of angiotensin-converting enzyme inhibitors (adjusted OR 1 · 11, 95% CI: 1 · 01–1 · 21), analysed for comparison reasons. There was a significant decreased risk associated with both severe acute pancreatitis, (OR 0 · 71 (0 · 59–0 · 85), and mild acute pancreatitis; adjusted OR 0 · 81 (0 · 70–0 · 94).

**Conclusion:**

This population-based case–control study indicates that use of angiotensin II receptor blockers might be associated with a lesser risk of acute pancreatitis, and that the protective association was significant among cases of both severe and mild acute pancreatitis.

**Electronic supplementary material:**

The online version of this article (doi:10.1186/s12876-017-0595-8) contains supplementary material, which is available to authorized users.

## Background

Pharmacological prevention of acute pancreatitis is warranted. Acute pancreatitis accounts for considerable morbidity and health care costs in Western countries. The reported incidence ranges between 5 and 70 per 100,000 persons per year, with an unexplained rising trend in several Western countries during the last decades [1–3]. The major risk factors, gallstone disease and excessive alcohol consumption, together account for about 2/3 of the cases [3–7]. Other risk factor include drugs, some viral infections and other uncommon reasons like endoscopic manipulation of the bile duct, leaving about 20% of the causes to the cases remain unexplained. A number of drugs have also been claimed to increase the risk of acute pancreatitis [8], e.g., valproate, [9] tetracycline, [10, 11] and 5-aminosalicylic acid [12]. Pharmacological prevention has been studied, especially of acute pancreatitis caused by endoscopic retrograde cholangio-pancreatography (ERCP), but without clinical success [13]. Angiotensin II receptor blockers (ARB) are often used to treat hypertension and heart failure. A local renin-angiotensin system in the human pancreas has been mapped, [14, 15] as a system that could influence pancreatic physiology and pathophysiology [16, 17]. Experimentally, the ARB losartan reduces the inflammation in induced acute pancreatitis among rats [18–20]. Previously we have performed a small randomized clinical trial, investigating the effect of losartan on pancreatic hyperenzymemia following endoscopic retrograde cholangio-pancreatography [21]. However, in our study we saw no differences between the groups, but the conclusion was hampered by lack of statistical power.

Our group performed a nested case–control study within a cohort of hypertension patients in the United Kingdom, suggesting a non-significant risk reduction of acute pancreatitis among users of ARB [22]. Also, cardiovascular disease per se increased risk of acute pancreatitis in a previous report [23].

## Methods

Our aim in this study was to elucidate the association between ARB with regard to risk of acute pancreatitis, by degree of severity, in a population-based case–control study, taking into account the inherent risk increase that these drug users have due to the indication of the anti-hypertensive medication.

### Study design

A Swedish nationwide, population-based case–control study was performed during January 1^st^ 2006 to December 31^st^ 2008. The source population was defined as all Swedish residents aged 40 to 84 years during the study period. Data on emigration, cancer and death was used for censoring to restrict the study cohort, and was obtained from the Register of the Total Population, the Cancer Register and the Causes of Death Register, respectively. We performed our case–control study nested in this study cohort. The Swedish Patient Register was used to identify cases of acute pancreatitis and provided data on comorbidity (concomitant diseases, defined below). The Register of the Total Population was used to select controls from the general population [24]. The resulting patients constituted the study population used in all subsequent analyses. Individual data on drug exposure among cases and controls was collected from the Prescribed Drug Register in Sweden [25]. Information about highest achieved formal educational level was received from the National Education Register The Swedish personal identity number, a unique 10-digit figure assigned to all Swedish residents, were used to link all study participants in the registers [26]. The Regional Ethical Review Board in Stockholm, Sweden approved the study.

### Patient register

The Patient Register [27] comprises information on all in-hospital care and outpatient specialist care in Sweden, including codes for diagnoses and surgical procedures. The Patient Register contains main and secondary discharge diagnoses registered according to the International Statistical Classification of Diseases and Related Health Problems [ICD]) codes for each occasion of in-hospital care, and has a complete nationwide coverage of in-patient data since January 1, 1987, and complete outpatient specialist care data since 2001. The positive predictive value of having a coding of acute pancreatitis in the Patient Register was 98% according to a recent validation study from our group, [28] thus indicating good validity of this diagnosis in the used register. All hospitalizations and episodes of care were registered with regard to length of stay, which was used as a proxy for severity of pancreatitis, based on established criteria for acute pancreatitis [29, 30].

### Prescribed drug register

From 1^st^ of July 2005 the Prescribed Drug Register includes the personal identity number and detailed information on all drugs that are prescribed and dispensed from all pharmacies in Sweden. In the register, information on each prescription includes strength and amount of the drug. Moreover, the register notes the drug’s dispensing date, and a free text section where the daily dose is recorded. The unique drug substance is recorded according to the Anatomical, Therapeutic, Chemical (ATC) classification [31].

### Case and control identification

Cases were defined as persons in the study cohort having a first time acute pancreatitis as a discharge diagnosis, according to the 10^st^ revision of the ICD, i.e., K85, in the Patient Register during 2006 to 2008. Cases were further subdivided by etiology: acute pancreatitis related to:

1. Alcohol (alcohol related disease, before or at index occasion (291, 303, 305A, 357 F, 425 F, 535D, 571A, 571B, 571C, 571D, or 980 in the Swedish version of ICD-9, or E244, F10, G312, G621, G721, I426, K292, K70, K852, O354, or T51 in ICD-10, or drugs used to treat alcohol dependence (ATC code N07BB).
2. Biliary disorders (ICD-10 K85.1 or having a gallstone related disease (ICD-10 K80, K81) or surgical gallbladder removal recorded at index occasion (ICD-10 procedural codes JKA20-21), and not alcohol pancreatitis.
3. Other type of pancreatitis, neither 1 nor 2.

Control subjects were randomly selected from the general population according to the principle of frequency-based density sampling, matched for age, sex, and calendar year using the Register of the Total Population. Medical conditions and surgical procedures were assessed through recorded hospitalizations or outpatient visits according to the Patient Register since 1987 or dispensed prescription since July 1st 2005. Medical drug exposure was related to an index date assigned to all study subjects. For cases, index date was set to be the date of admission for acute pancreatitis. For controls, the index date was the randomly assigned date within the study period. Cases and eligible controls with a previous cancer, apart from non-melanoma cancers of the skin, or any pancreatic disease (K85, K86, K87 [ICD-10], and 577 [ICD-9]) recorded in the Patient Register before the study started were excluded.

### Exposure to angiotensin II receptor blockers

Dispensed drug prescriptions of ARB were identified by their specific ATC codes (C09C, C09D) in the Prescribed Drug Register. Only exposure prior to index date was considered in the study. We assumed that a prescription normally lasted 114 days of drug usage, allowing a 14-day margin for prescription renewal. Drug exposure was defined as *current* or *past* if the drug had been dispensed from 0 to 114, and 115–180 days, respectively in relation to index date. The absence of a prescription or prescription > 180 days before index date was classified as *non*-*use*.

The duration of ARB usage was analysed in the following way: current users was based on the number of days elapsed since the first prescription of ARB. Duration was divided into recent user, first prescription less than 90 days; 3–6 months; 6–12 months, and past usage.

### Statistical analysis

The relative risk of acute pancreatitis was estimated by unconditional logistic regression, used to calculate odds ratios (OR) with 95% confidence interval (CI). The selection of variables was partially based on a priori decision regarding known risk factors as potential confounding factors, i.e. gallstone disease, alcohol related disease, level of education, type 2 diabetes and chronic obstructive pulmonary disease (as a proxy for smoking). Also, to evaluate the potential for confounding by indication, we added presence of cardiovascular disorder, as defined in a previous paper ([23]). In addition, added a comorbidity index, based on number of distinct medications, defined below.

This resulted in three different models that were analysed by the logistic regression. First, model 1 included the matching variables i.e. sex, age (in categories 40–49, 50–59, 60–69, 70–79, 80–84 years) and calendar year. Next, presence of any cardiovascular disorder (CVD) [23] (Yes or No) was introduced to act as a proxy for severity of indication for ARB and ACE-inhibitors, named Model 2. Finally, the following variables were included in the full model 3: 1) a general co-morbidity index measured by number of distinct medications, [23, 32] a score defined as the sum of unique ATC codes (based on the first 7 digits), dispensed during the last 6 months from index date, and divided into categories (0–1, 2–4, 5–9, 10–14, and ≥15 prescriptions). 2) Education level divided into three categories of highest attained education: elementary school, secondary school, university studies, and one category for missing data. 3) Alcohol related diseases present before index date (see ICD codes above) 4) Gallstone disease recorded before index date, see specification of ICD codes. 5) Chronic obstructive pulmonary disorder (491, 492, or 496 in ICD-9, or J41, J42, J42, J44 in ICD-10). 7) Type 2 diabetes (250 in ICD9, or E10-E14 in ICD10, or anti-diabetic medication (ATC code A10). To further assess the effect of confounding by indication, we separately analysed the association angiotensin-converting enzyme (ACE)-inhibitors (ATC codes C09A and C09B) and acute pancreatitis. The severity of pancreatitis was classified based on number of days in-hospitalised: 1–3 days, 4–7, and >7 corresponding to mild, moderate, and severe form of acute pancreatitis, respectively. Moreover, we made a sub-analysis on any use of anti-hypertensive drugs based on ATC-codes; ARB, ACE-inhibitors, beta-blockers (C07), calcium channel blockers (C08), and diuretics (C03).

The statistical software Stata®, version 11 (StataCorp, Texas, USA) was used for statistical analysis.

## Results

### Study participants

During follow-up, 6,161 cases of first-time acute pancreatitis were identified, rendering a crude incidence of 22 per 100,000 person-years (data not shown). From the study cohort, 61,637 persons were randomly selected as controls. Some characteristics of the study participants are described in Table 1. Men were slightly overrepresented among cases of acute pancreatitis. The ratio of university education was slightly lower among cases compared to controls. The median number of medications was higher among ARB users, 5 vs. 4 among controls (data not shown).

**Table 1:** Basic characteristics among case patients with acute pancreatitis and matched control persons

Exposure	Controls	Cases
	*N* (%)*	*N* (%)*
Total	61,637 (100)	6,161 (100)
Calendar year		
2006	19,963 (32)	1,996 (32)
2007	20,728 (34)	2,071 (34)
2008	20,946 (34)	2,094 (34)
Sex		
Male	33,892 (55)	3,387 (55)
Female	27,745 (45)	2,774 (45)
Age		
40–49	10,562 (17)	1,055 (17)
50–59	13,978 (23)	1,397 (23)
60–69	16,590 (27)	1,659 (27)
70–79	13,908 (23)	1,391 (23)
80–84	6,599 (11)	659 (11)
Education level		
Elementary school	21,896 (36)	2,464 (40)
Secondary school	24,016 (39)	2,455 (40)
University studies	14,769 (24)	1,099 (18)
Missing data	956 (2)	143 (2)
Alcohol related disease	1,549 (3)	682 (11)
Gallstone disease	2,595 (4)	2,246 (36)
Chronic obstructive pulmonary disease	1,328 (2)	326 (5)
Number of distinct medications		
0–1	25,373 (41)	1,468 (24)
2–4	16,953 (28)	1,665 (27)
5–9	13,656 (22)	1,851 (30)
10–14	4,252 (7)	781 (13)
≥ 15	1,403 (2)	396 (6)

*Percentages were rounded; hence the sum could be more or less than 100

### Angiotensin II receptor blockers and risk of acute pancreatitis

The association between exposure to angiotensin II receptor blockers and acute pancreatitis is displayed in Table 2. Current users of angiotensin II receptor blockers had an elevated OR of 1 · 18 (95% CI 1 · 08–1 · 30) of acute pancreatitis in the crude model with adjustment for matching variables. In the next step, adjusting for underlying CVD, the risk was estimated to be decreased; OR = 0 · 82, 95% CI 0 · 74–0 · 90. Moreover, usage of angiotensin II receptor blockers showed a decreased OR of 0 · 77 (95% CI 0 · 69–0 · 86) for acute pancreatitis, compared to non-users adjusted for the full model 3 (Table 2). Also, the results regarding type of ARB and risk of acute pancreatitis is shown in Additional file 1: Table S1A.

**Table 2:** Exposure to angiotensin II receptor blockers (ARB), angiotensin-converting enzyme (ACE) inhibitors and their relative risk for acute pancreatitis estimated by odds ratio (OR) with 95% confidence intervals (CI)

Exposure	Controls	Cases	Model 1^a^	Model 2^b^	Model 3^c^
	*N* (%)	*N* (%)	OR 95% CI	OR 95% CI	OR 95% CI
Total	61,637 (100)	6,161 (100)			
ARB					
Never use	56,542 (92)	5,571 (90)	1 (reference)	1 (reference)	1 (reference)
Current use	4,715 (8)	548 (9)	1 · 18 (1 · 08–1 · 30)	0 · 82 (0 · 74–0 · 90)	0 · 77 (0 · 69–0 · 86)
Past use	380 (1)	42 (1)	1 · 13 (0 · 82–1 · 55)	0 · 77 (0 · 74–0 · 90)	0 · 70 (0 · 49–1 · 01)
ACE-inhibitors					
Never use	55,073 (89)	5,080 (82)	1 (reference)	1 (reference)	1 (reference)
Current use	6,067 (10)	987 (16)	1 · 82 (1 · 69–1 · 96)	1 · 16 (1 · 07–1 · 26)	1 · 11 (1 · 01–1 · 21)
Past use	497 (1)	94 (2)	2 · 11 (1 · 69–2 · 63)	1 · 37 (1 · 09–1 · 72)	1 · 24 (0 · 95–1 · 60)

^a^Adjusting for sex, age and calendar year. ^b^As Model 1 + presence of cardiovascular disorder

^c^Adjusting for sex, age, calendar year, education, alcohol related disease, gallstone disease, chronic obstructive pulmonary disease, diabetes, cardiovascular disorder, and number of distinct medications, Angiotensin-converting enzyme (ACE)-inhibitors and ARB

We have analysed the effect of duration of ARB usage and found that current users with more than 6 months of usage, had lower OR of 0.77 with 95% CI 0.70–0.86, compared to recent users with an OR of 1.04, 95% CI 0.68–1.58 (Additional file 1: Table S1B-C).

Table 3 presents the association between ARB and the severity of pancreatitis. Notably, the risk was reduced for all severities of acute pancreatitis, e.g. the risk for severe acute pancreatitis was significantly reduced among ARB users with a fully adjusted OR 0 · 71, 95% CI 0 · 59–0 · 85, compared to non-users.

**Table 3:** Exposure to angiotensin receptor blockers (ARB) and severity of acute pancreatitis, estimated by odds ratios (OR) with 95% confidence intervals (CI)

Exposure	Controls	Mild acute pancreatitis	Moderate acute pancreatitis	Severe acute pancreatitis
	*N* (%)	*N* (%)	*N* (%)	*N* (%)
Total, *n* (%)	61,637 (100)	2,783 (100)	1,542 (100)	1,814 (100)
ARB	56,542 (92)	2,514 (90)	1,403 (91)	1,632 (90)
Never use				
Current use	4,715 (8)	249 (9)	131 (8.5)	168 (9)
* Model 1 OR* (*95* % *CI*)	1 (Reference)	1 · 25 (1 · 10–1 · 44)	1 · 08 (0 · 90–1 · 30)	1 · 18 (1 · 00–1 · 39)
* Model 2* (*OR* (*95* % *CI*)		0 · 86 (0 · 75–0 · 99)	0 · 73 (0 · 61–0 · 88)	0 · 76 (0 · 64–0 · 89)
* Model 3 OR* (*95* % *CI*)		0 · 81 (0 · 70–0 · 94)	0 · 71 (0 · 58–0 · 87)	0 · 71 (0 · 59–0 · 85)
Past use	380 (1)	20 (0.5)	8 (0.5)	14 (1)
* Model 1 OR* (*95* % *CI*)	1 (Reference)	1 · 24 (0 · 79–1 · 95)	0 · 82 (0 · 41–1 · 66)	1 · 23 (0 · 72–2 · 10)
* Model 2 OR* (*95* % *CI*)		0 · 85 (0 · 54–1 · 33)	0.56 (0.28–1.13)	0 · 79 (0 · 49–1 · 35)
* Model 3 OR* (*95* % *CI*)		0 · 75 (0 · 46–1 · 22)	0.53 (0 · 26–1 · 08)	0 · 75 (0 · 42–1 · 29)

1) Adjusting for sex, age and calendar year

2) Adjusting for sex, age, calendar year and presence of cardiovascular disorder

3) Adjusting for sex, age, calendar year, education, chronic obstructive pulmonary disease, diabetes, alcohol related disease, cardiovascular disorder, and number of distinct medications

Stratifying acute pancreatitis by etiology resulted in similarly negative association between angiotensin II receptor blockers and gallstone related, (OR = 0 · 67, 95% CI: 0 · 57–0 · 78) alcohol related (OR = 0 · 50, 95% CI:0 · 37–0 · 68), and other acute pancreatitis (OR = 0 · 77, 95% CI :0 · 67–0 · 88) (Additional file 2: Table S2).

By restricting the analysis to only users of cardiovascular drugs there was a significant risk decrease, adjusting for matching factors (OR 0 · 81, 95% CI 0 · 74–0 · 90), and in the fully adjusted model (OR 0 · 84, 95% CI 0 · 75–0 · 92). (Additional file 2: Table S3).

### Exposure to angiotensin-converting enzyme inhibitors and risk of acute pancreatitis

Current use of ACE inhibitors was associated with an increased OR of acute pancreatitis in the full multi-variable adjustment (OR = 1 · 11, 95% CI: 1 · 01–1 · 21) (Table 2). In the Additional file 2: Table S3, we analysed the effect of ACE usage within the cohort of cardiovascular/anti-hypertensive medications, and noticed an increased risk (adjusted OR 1 · 31, 95% CI 1 · 21–1 · 43).

## Discussion

This study suggests that usage of ARB might be associated with a lower risk of acute pancreatitis.

ARB users had more comorbidity than non-users, such as cardiovascular disorders, which entail an increased risk, [23] acting as a potential confounding factor. This could explain why the risk estimate adjusting for only the matching variables was in fact increased. However, we have scrutinized this connection in several ways; first, after adjusting for cardiovascular disorders, which have been shown to increase risk of acute pancreatitis in a previous paper [23]. After this adjustment, which was a way of taking confounding by indication into account, the association between ARB and acute pancreatitis was negative. Thus, indicating a potential protective effect for ARB. From the outset, we expected comorbidity in general to be a potential confounding factor relying on previous research and therefore included a comorbidity index, based on number of individual medications [23, 32]. Number of distinct medications had a large impact on the estimated relative risk of acute pancreatitis both in the stratified analysis (data not shown), and as a covariate in the multivariable regression model *indicating the importance of comorbidity as a confounding facto*r. Second, we performed an analysis restricted to users of any cardiovascular/antihypertensive drug, and in this subsample looked into the association between ARB and acute pancreatitis. The protective association was present in the model adjusting for matching variables, suggesting that in comparison to other hypertensive patients prescribed other drugs, the relative risk of ARB was decreased in contrast to the increased risk associated with ACE-inhibitors. (Additional file 2: Table S3).

Additionally, to evaluate the risk of confounding by indication, we compared the result for ARB with ACE-inhibitors. ACE-inhibitors have similar indications, but for which we did not hypothesize a protective relationship of acute pancreatitis. In the full multi-variable model a positive association for ACE-inhibitors was found. However, ARB use was negatively associated with acute pancreatitis after adjustment in the full multi-variable model. Hence, this supports the main finding of a separate and potentially protective association between ARB and acute pancreatitis, and also reduces the risk of confounding by indication.

Also, when we evaluated the effect of degree of severity of pancreatitis, as shown in Table 3, the effect of ARB was seen across all severities of acute pancreatitis.

The duration of ARB use seemed to be important as indicated by the lower risk seen in users with more than 6 months of usage. We believe that the effect of ARB usage is a long-term effect reducing inflammation and thereby increasing the threshold of initiating an episode of pancreatitis. Regarding dosage, our data set did not include such data, but subsequent studies need to investigate this issue.

Previously, we carried out a study in a primary care cohort of hypertensive patients in the United Kingdom, [22] which suggested a statistically non-significant decreased risk of acute pancreatitis in subjects exposed to angiotensin II receptor blockers, but not among users of other anti-hypertensive medications. The sample size of that study was smaller and the hypertension cohort was different compared to the present report. The main advantages of the present study were the large sample-size, and the population-based design reducing chance errors and selection bias, respectively. The Prescribed Drug Register and the Patient Register gave unbiased access to the study exposure, outcome and some potential confounders. The validity of the outcome measurement was high, according to our recent validation study [28].

Some potential drawbacks warrant further comments. A common problem in observational studies is lack of precision in measuring exposure. Our cut-off for current use of 114 days was chosen based on the mean length of a prescription of 100 days, allowing a 2 weeks margin for renewal of prescription to index date. To address the potential misclassification of exposure, two alternative approaches were used. First, the cut-off was changed to 90 days for current use. Second, information on dosage from the manual review was used to calculate the actual prescription length in relation to index date. Neither of the two approaches changed the association substantially between angiotensin II receptor blocker and acute pancreatitis (data not shown). Still, some misclassification of exposure could remain, contributing to the non-significant decreased risk seen among past users. Another potential weakness is incorrect handling of confounding factors. Alcohol consumption was measured indirectly, i.e. based on diagnosis of alcohol related disorders in the present report. In a recent study [28] from our group validating the acute pancreatitis diagnosis and classifying the type of pancreatitis after a manual chart review, we found a similar ratio of alcohol related pancreatitis using our proxy variable; alcohol related disease. This suggests that our measurement of alcohol exposure is reliable. We also adjusted for the usage of anxiolytics, which is associated with high alcohol consumption. This adjustment did not affect the OR of ARB and acute pancreatitis (data not shown). In addition, to evaluate the potential confounding effect of tobacco smoking, an established risk factor of acute pancreatitis, [33] we adjusted for chronic obstructive pulmonary disease, a disease closely related to tobacco smoking. This adjustment did not substantially affect the risk estimate of ARB and acute pancreatitis, why tobacco smoking was not considered an important confounding factor for this association.

Furthermore, confounding by socioeconomic position could be present, as there has previously been described a lower exposure to angiotensin II receptor blockers in low educated groups [34]. However, adjusting for socioeconomic position did not substantially affect our risk estimates contradicting such confounding.

Angiotensin II has been proposed to be a pro-inflammatory mediator, [35] hence blocking the main receptor pathway would be beneficial. Angiotensin II receptor blockers could also reduce ischemia and consequently inflammation [16]. Recently losartan showed reduced biomarker level for pancreatic inflammation in an experimental model [36]. Although poorly understood in the human pancreas, this could explain our protective finding, in addition to animal studies on experimentally induced pancreatitis [18–20, 37].

## Conclusions

In conclusion, this Swedish population-based case–control study suggests a protective effect of angiotensin II receptor blockers on the risk of developing acute pancreatitis. Further studies are warranted before a causal association can be established.

## Additional files

Additional file 1: Table S1A. Type of Angiotensin Receptor Blockers in relation to acute pancreatitis status. **Table S1B.** Duration of Angiotensin Receptor Blockers usage in relation to acute pancreatitis status. **Table S1C.** Estimated risk of acute pancreatitis in relation to duration of ARB. (DOCX 19 kb)
Additional file 2: Table S2. Exposure to angiotensin receptor blockers (ARB) and risk of different types of acute pancreatitis, estimated by odds ratios (OR) with 95% confidence intervals (CI), in a nested case–control study in Sweden. **Table S3.** Exposure to angiotensin receptor blockers (ARB) stratified among users of cardiovascular drugs, estimated by odds ratios (OR) with 95% confidence intervals (CI). (DOCX 20 kb)

## Abbreviations

ACE: Angiotensin I-converting enzyme
ARB: Angiotensin II receptor blockers
ATC: Anatomic therapeutic classification
CI: Confidence interval
ERCP: Endoscopic retrograde cholangio-pancreatography
ICD: International statistical classification of diseases and related health problems
OR: Odds ratio
