# Supplementary material for: Angiotensin II receptor blockers and risk of acute pancreatitis - a population based case–control study in Sweden
Source: BMC Gastroenterol. 2017 Mar 7;17:36. doi: 10.1186/s12876-017-0595-8 (PMC5341438; doi:10.1186/s12876-017-0595-8)
Supplement: Additional file 1: Table S1A. — Type of Angiotensin Receptor Blockers in relation to acute pancreatitis status. Table S1B. Duration of Angiotensin Receptor Blockers usage in relation to acute pancreatitis status. Table S1C. Estimated risk of acute pancreatitis in relation to duration of ARB. (DOCX 19 kb) [file 12876_2017_595_MOESM1_ESM.docx]

| **Table S1A. Type of Angiotensin Receptor Blockers in relation to acute pancreatitis status** | | | | |
| --- | --- | --- | --- | --- |
| **Type of ARB** | **Controls** | **Case** | **Total** |  |
|  |  |  |  |  |
| **Total n** | 56,542 | 5571 | 62,113 |  |
| Percentage | 91,7 | 90,42 | 91,6 |  |
|  |  |  |  |  |
| Losartan n | 1,779 | 208 | 1,987 |  |
| % | 2,9 | 3.4 | 2,8 |  |
|  |  |  |  |  |
| Eprosartan n | 42 | 2 | 44 |  |
| % | 0,1 | 0 | 0,1 |  |
|  |  |  |  |  |
| Valsartan n | 499 | 55 | 554 |  |
| % | 0,8 | 0,9 | 0,8 |  |
|  |  |  |  |  |
| Irbesartan n | 553 | 69 | 622 |  |
| % | 0,9 | 1,1 | 0,9 |  |
|  |  |  |  |  |
| Candesartan n | 1,760 | 207 | 1,967 |  |
| % | 2,9 | 3,4 | 2,9 |  |
|  |  |  |  |  |
| Telmisartan n | 82 | 7 | 89 |  |
| % | 0,1 | 0,1 | 0,1 |  |
|  |  |  |  |  |
| Past use n | 380 | 42 | 422 |  |
| % | 0,6 | 0,7 | 0,6 |  |
|  |  |  |  |  |
| Total n | 61,637 | 6161 | 67,798 |  |
| % | 100 | 100 | 100 |  |
| Overall significance testing showing p-value less than 0.05 (chi2-test) | |  |  |  |

**Additional file 1:**

| **Table S1B. Duration of Angiotensin Receptor Blockers usage in relation to acute pancreatitis status** | | | | | | | | | |
| --- | --- | --- | --- | --- | --- | --- | --- | --- | --- |
|  |  | | |  | |  | | | |
|  | |  |  | |  | |  |  |  |
| Status of ARB use and duration | |  | Non-case Case | |  | | Total |  |  |
|  | |  |  | |  | |  |  |  |
| Non-use | | n | 56,542 | | 5,571 | | 62,113 |  |  |
|  | | % | 91.7 | | 90.4 | | 91.6 |  |  |
|  | |  |  | |  | |  |  |  |
| Recent user <90 days | | n | 165 | | 26 | | 191 |  |  |
|  | | % | 0.3 | | 0.4 | | 0.28 |  |  |
|  | |  |  | |  | |  |  |  |
| 3-6 months | | n | 604 | | 70 | | 710 |  |  |
|  | | % | 1.0 | | 1.1 | | 1.1 |  |  |
|  | |  |  | |  | |  |  |  |
| 6-12 months | | n | 3,91 | | 452 | | 4,362 |  |  |
|  | | % | 6.3 | | 7.3 | | 6.4 |  |  |
|  | |  |  | |  | |  |  |  |
| Past users | | n | 380 | | 42 | | 422 |  |  |
|  | | % | 0.6 | | 0.7 | | 0.6 |  |  |
|  | |  |  | |  | |  |  |  |
| Total | | n | 61,637 6,161 | | 6,161 | | 67,798 |  |  |
|  | | % | 100 | | 100 | | 100 |  |  |
| p-value less than 0.05 (chi2-test) | | | | |  | |  |  |  |

| **Table S2C. Estimated risk of acute pancreatitis in relation to duration of ARB** | | | | |
| --- | --- | --- | --- | --- |
|  | Odds ratio | p-value | 95% CI* |  |
| Non-usage | 1.00 | N/A | Reference |  |
| Recent user <90 days | 1.039761 | 0.86 | 0.68 | 1.58 |
| 3-6 months usage | .7334255 | <0.05 | 0.57 | 0.92 |
| 6-12 months | .7728741 | <0.05 | 0.70 | 0.86 |
| *Confidence interval |  |  |  |  |
