# Supplementary material for: Angiotensin II receptor blockers and risk of acute pancreatitis - a population based case–control study in Sweden
Source: BMC Gastroenterol. 2017 Mar 7;17:36. doi: 10.1186/s12876-017-0595-8 (PMC5341438; doi:10.1186/s12876-017-0595-8)
Supplement: Additional file 2: Table S2. — Exposure to angiotensin receptor blockers (ARB) and risk of different types of acute pancreatitis, estimated by odds ratios (OR) with 95% confidence intervals (CI), in a nested case–control study in Sweden. Table S3. Exposure to angiotensin receptor blockers (ARB) stratified among users of cardiovascular drugs, estimated by odds ratios (OR) with 95% confidence intervals (CI). (DOCX 20 kb) [file 12876_2017_595_MOESM2_ESM.docx]

# Additional file 2:

| **Table S2. Exposure to angiotensin receptor blockers (ARB) and risk of different types of acute pancreatitis, estimated by odds ratios (OR) with 95% confidence intervals (CI), in a nested case-control study in Sweden.** | | | | |
| --- | --- | --- | --- | --- |
| Exposure | Controls | Gallstone related pancreatitis | Alcohol related pancreatitis | Other acute pancreatitis |
|  | N (%) | N (%) | N (%) | N (%) |
| Total, n (%) | 61,637 (100) | 2,393 (100) | 816 (100) | 2,952 (100) |
| ARB  Never use | 56,542 (92) | 2,178 (91) | 760 (93) | 2,633 (89) |
| Current use | 4,715 (8) | 200 (8) | 51 (6) | 297 (10) |
| *Model 1 OR (95% CI)* | 1 (Reference) | 1·08 (0·93-1·25) | 1·06 (0·79-1·41) | 1·30 (1·14-1·47) |
| *Model 2 OR (95% CI)* | 1  (Reference) | 0·67 (0·57-0·78) | 0·50 (0·37-0·68) | 0·77 (0·67-0·88) |
| Past use | 380 (1) | 15 (1) | 5 (1) | 22 (1) |
| *Model 1 OR (95% CI)* | 1 (Reference) | 1·00 (0·59-1·66) | 1·23 (0·51-3·00) | 1·21 (0·79-1·87) |
| *Model 2 OR (95% CI)* |  | 0·63 (0·38-1·07) | 0.55 (0·22-1·37) | 0.68 (0·44-1·07) |
| 1) Adjusting for sex, age and calendar year.  2) Adjusting for sex, age, calendar year, education, chronic obstructive pulmonary disease, diabetes, cardiovascular disorder, and number of distinct medications. | | | | |

| **Table S3. Exposure to angiotensin receptor blockers (ARB) stratified among users of cardiovascular drugs, estimated by odds ratios (OR) with 95% confidence intervals (CI)** | | | | |
| --- | --- | --- | --- | --- |
| **Exposure** | **Controls using cardiovascular drugs** | **Cases using cardiovascular drugs** | ***Model 1 OR (95% CI)*** | ***Model 2 OR (95% C*** |
|  | **N (%)** | **N (%)** |  |  |
| Total, n (%) | 20,908 (100) | 2,829 (100) |  |  |
| **ARB** |  |  |  |  |
| Never use | 16,039 (77 | 2,255 (80) | 1 (Reference) | 1 (Reference) |
| Current use | 4,715 (23) | 548 (19) | 0·81 (0·74-0·90) | 0·84 (0·75-0·92) |
|  |  |  |  |  |
| Past use | 154 (1) | 26 (1) | 1.18 (0.78-1.79) | 1.04 (0·68-1·49) |
|  |  |  |  |  |
| **ACE-inhibitors** |  |  |  |  |
| Never use | 14,578 (70) | 1,792 (63) | 1 (Reference) | 1 (Reference) |
| Current use | 6,067 (29) | 987 (35) | 1·31 (1·21-1·43) | 1·23 (1·12-1·34) |
| Past use | 263 (1) | 50 (2) | 1·58 (1·16-2·14) | 1·34 (0·98-1·83) |
| 1) Adjusting for sex, age and calendar year   2) Adjusting for sex, age, calendar year, education, alcohol related disease, chronic obstructive pulmonary disease, diabetes, and number of distinct medications, ACE use and ARB as also included | | | | |
